# Literary Notes

**Published:** 1909-06-26

**Authors:** 


### LITERARY NOTES.

We have received from the National Food Reform Association, 40 Chandos Street, Charing Cross, London (temporary address), a booklet containing an unpublished memorandum on "The Dietetic Treatment of Inebriety," submitted by it to the recent Departmental Committee, as well as important letters from the Dean of Durham, Sir Thomas Barlow, Mrs. Bramwell Booth, the Rev. Dr. Paton, Drs. Haig and Kellogg. A specimen copy will be sent post free by the Secretary on the receipt of three penny stamps.

We learn that the friends of the late Mr. H. L. Barnard, F.R.C.S., whose untimely death, last year, at the age of forty-one, was deplored by the London Hospital in particular, and by the surgical world in general, have decided to publish as a memorial his entire contributions on Abdominal Surgery in book form. In 1900 Mr. Barnard projected this volume of collected surgical papers, and two former pupils, Dr. F. Wood-Jones and Mr. Stanley Beale, undertook the task of illustration. The editorship of this work, which was almost finished when death intervened, is in the hands of the late author's colleague, Mr. James Sherren, and Dr. H. H. Bashford will contribute the biography. The cost of publication, etc., is estimated at £200, and the Memorial Committee appeals to Mr. Barnard's friends and pupils for subscriptions, which should be sent to the Hon. Secretary and Treasurer, Dr. Cecil Wall, 6 Cavendish Place, W. We are certain that this volume will possess merits, intrinsic and extrinsic, which should ensure for it a very wide circulation.

A new edition of Dr. Burton-Fanning's "Open-Air Treatment of Pulmonary Tuberculosis" (Modern Methods of Treatment Series) is announced by Cassell and Co., Ltd. In this edition the author deals with the researches of Sir A. E. Wright and his collaborators, as well as with the new methods of early diagnosis, including Calmette's opthalmo-reaction.

We have received from the publishers, 30 Fleet Street, London, E.C., a copy of "Holidays Abroad," an illustrated booklet to a series of tours in less-known districts of Holland, North Germany, the side valleys of the Rhine, the Belgian Ardennes, and the Tyrol, easily and inexpensively reached by the Great Eastern Railway Company's Harwich route to the Continent.

